# Stercoral re-perforation after colostomy takedown: a case report

**DOI:** 10.1186/s12893-021-01118-1

**Published:** 2021-03-09

**Authors:** Seunghwan Lee, Chang Woo Kim

**Affiliations:** Department of Surgery, Kyung Hee University Hospital At Gangdong, Kyung Hee University School of Medicine, 892 Dongnam-ro, Gangdong-gu, 05278 Seoul, South Korea

**Keywords:** Stercoral perforation, Re-perforation, Colostomy, Stoma takedown

## Abstract

**Background:**

Stercoral perforation (SP) is a rare surgical condition that is associated with high morbidity and mortality. Most of these patients undergo emergent surgery, including colostomy, and some undergo colostomy takedown after recovery. Stercoral re-perforation after colostomy takedown followed by colostomy for SP has not yet been reported.

**Case presentation:**

A 79-year-old woman presented with abdominal pain for one day. Abdominal-pelvis computed tomography revealed pneumoperitoneum with diffuse mesenteric fat haziness of the left abdomen. During laparoscopic exploration, a 3-cm-sized perforated site was found at the sigmoid-descending colon, with fecal material and reactive fluid outside the colon. Loop colostomy formation was performed, and a takedown was completed after 3 months. Two years 4 months after the initial procedure, the patient was re-admitted to our hospital with abdominal pain. She underwent a second laparoscopic colostomy formation and was discharged, although the postoperative clinical course was poorer than that after the first surgery.

**Conclusions:**

This case of stercoral re-perforation after colostomy takedown followed by colostomy formation for SP has important clinical implications and can be a reference for physicians. When the first colostomy formation was performed for SP, the decision on performance of a colostomy takedown should be made after carefully considering several factors.

## Background

Stercoral perforation (SP) is a rare life-threatening condition with poor prognosis. Increased intraluminal pressure due to chronic constipation or fecal impaction has been considered an etiology of SP [1–3]. Accurate diagnosis can be difficult when free air is observed in the intraperitoneal space without a definite perforation site, although imaging studies have improved over the last decade [2]. Moreover, SP is not well-differentiated from other causes of perforation such as diverticulitis or malignancy [4].

When SP is suspected, one of various types of surgery is needed [1]. Colostomy formation with or without perforated colon resection is performed in most cases, although resection of perforated colon and anastomosis might be considered in selective cases in which fecal spillage or soiling was not severe. After colostomy formation and recovery, many patients complain of discomfort and request a takedown. An appropriate decision regarding colostomy takedown should be made, and the logistics for each patient should be considered. We describe a case of stercoral re-perforation after colostomy takedown for SP.

## Case presentation

A 79-year-old female patient was admitted to the emergency department of Kyung Hee University Hospital at Gangdong, Seoul, Korea, complaining of abdominal pain that started one day prior. She was 150 cm tall and weighed 40 kg. She was on medication for diagnosed atrial fibrillation and had surgical history for uterine prolapse, rectal prolapse, and left femur neck fracture. Although the patient was not bedridden, she had spent most of her days in bed since undergoing left total hip replacement surgery two years prior. Her average frequency of defecation was every 3 days. Trauma or external injury was denied by the patient and her family.

At the emergency department, her body temperature was 36.7 ℃, blood pressure was 81/51 mmHg, heart rate was 106/min, and respiratory rate was 21 breaths/min. Whole abdominal distention and direct tenderness were noted. Laboratory examinations showed leukopenia (1.88 × 10^3^/uL), and her serum C-reactive protein was 1.1 mg/dL. Blood biochemistries and urine analysis were normal.

**Fig. 1:**
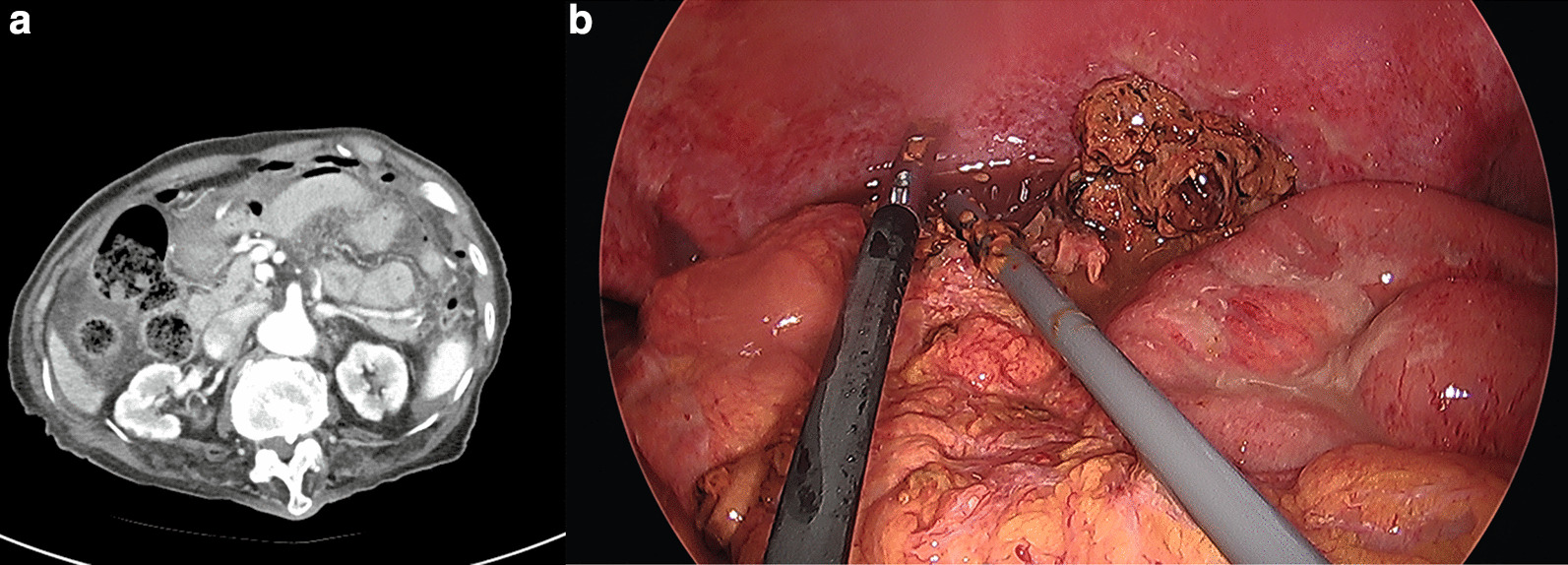
**a** Computed tomography (CT) scan of the patient. **b** Descending colon perforation was found during surgery

An abdominal-pelvis computed tomography (CT) scan revealed pneumoperitoneum with diffuse mesenteric fat haziness of the left abdomen (Fig. 1a). However, the perforated site could not be clearly delineated. Because the patient’s abdominal pain was aggravated and her blood pressure gradually decreased, she underwent emergency surgery without further evaluation.

Intravenous fluid resuscitation was performed in the emergency department. Second-generation cephalosporin and metronidazole were administered before emergency surgery. We performed laparoscopic surgery with the patient under general endotracheal anesthesia. The camera was inserted through an infraumbilical 12-mm trocar, and two 5-mm trocars were inserted at the right upper and lower quadrants. A 3-cm-sized perforated site was found at the antimesenteric border of the sigmoid-descending junction colon, with fecal material and reactive fluid outside the colon (Fig. 1b). In addition, a moderately dilated descending colon was found and was filled with hard feces. We mobilized the lateral detachment to create a colostomy using the perforation site (exteriorization) and formed a loop colostomy at the left upper quadrant. The patient developed ileus at postoperative day eight and recovered with conservative treatment including Levin tube insertion. No other events were observed, and she was discharged at postoperative day 29.

Three months after the emergency surgery, the patient visited the outpatient clinic and required colostomy repair surgery. Colonoscopy was performed to identify causes of perforation, such as diverticulum or malignancy, which were negative. The patient was given a detailed explanation about the high possibility of perioperative morbidity and mortality due to advanced age, underlying diseases, and poor performance status; however, she requested colostomy removal. We performed colostomy repair under general anesthesia, with hand-sewn end-to-end anastomosis. The patient was discharged at postoperative day 10 without any adverse events.

Two years 4 months later, the patient was re-admitted to our hospital with abdominal pain. The patient had spent most of her days bedridden due to general weakness; her average frequency of defecation was every 4 or 5 days.

On admission at the emergency department, her body temperature was 36.5 ℃, blood pressure was 91/62 mmHg, heart rate was 112/min, and respiratory rate was 20 breaths/min. Whole abdominal distention and direct tenderness were noted. Leukocytosis was identified (12.10 × 10^3^/uL), and predominant neutrophils (91%) with low hemoglobin (8.4 g/dL) were found. Serum C-reactive protein was increased at 8 mg/dL, and the erythrocyte sedimentation rate was 43 mm/h. Blood biochemistry showed hypoalbuminemia (2.7 g/dL) and hyponatremia (129 mEq/L), and the carcinoembryonic antigen (CEA) level was increased at 12.4 ng/mL.

**Fig. 2:**
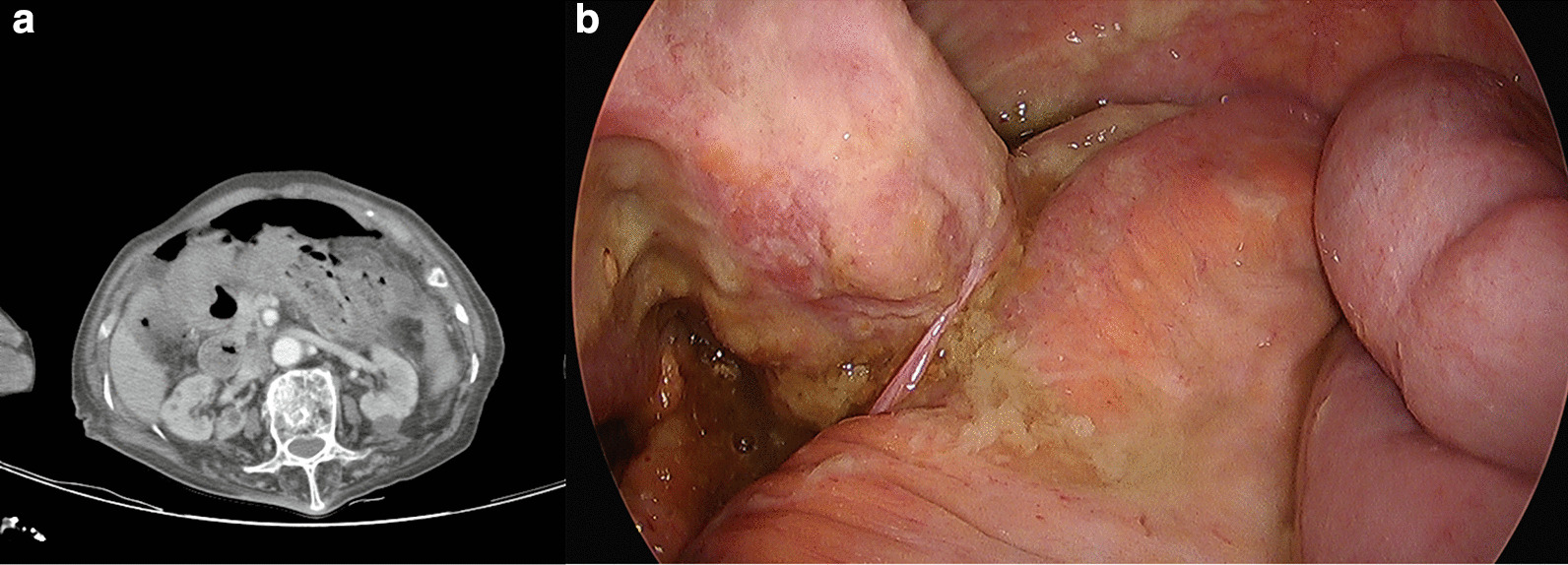
**a** CT scan of the patient. **b** Transverse colon perforation was found during surgery

An abdominal-pelvis CT scan revealed pneumoperitoneum and wall discontinuity in the transverse colon, with adjacent dirty fluid collection in the mid- to left side mesentery, suggesting distal transverse colon perforation with spillage of fecal material (Fig. 2a).

**Fig. 3:**
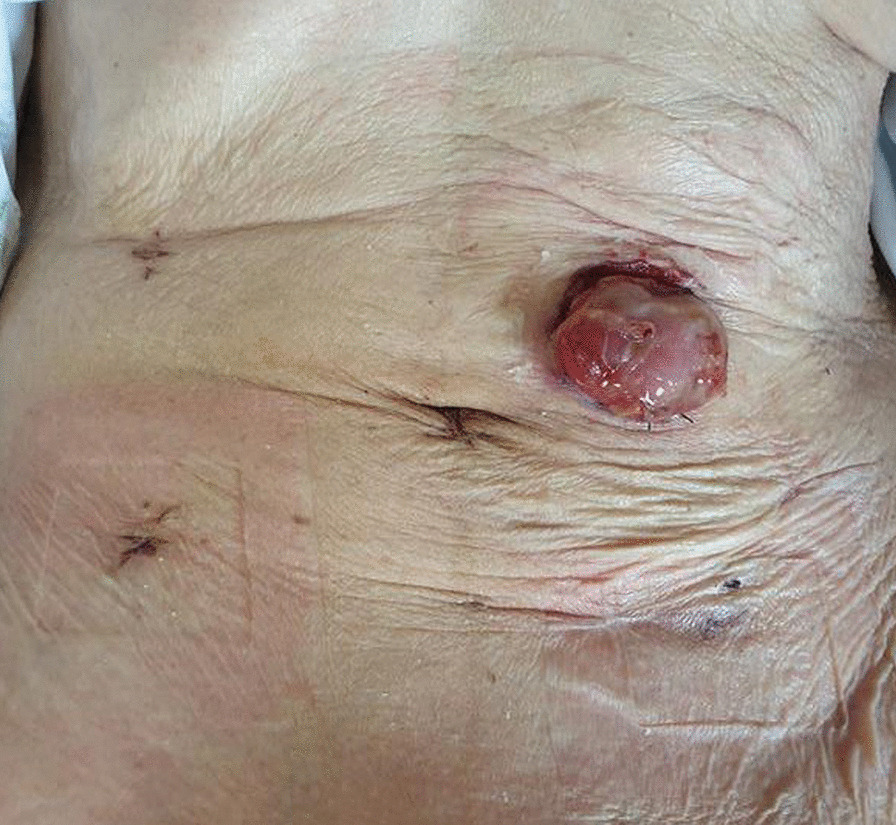
The patient’s operative wounds and colostomy

A treatment strategy similar to the previous procedure was applied. Intravenous fluid resuscitation was conducted, and second-generation cephalosporin and metronidazole were administered before emergency surgery. We performed another laparoscopic surgery under general endotracheal anesthesia. The camera and trocars were inserted through an infraumbilical approach via the right upper and lower quadrants. A 2-cm-sized perforation was found at the antimesenteric border of the distal transverse colon with fecal material, proximal to the previous perforation site (Fig. 2b). However, additional fecal material and dirty fluid were identified compared with the findings from 2 years prior. After the splenic flexure was completely mobilized, a loop colostomy was performed using the perforated site at the left upper quadrant (Fig. 3).

**Fig. 4:**
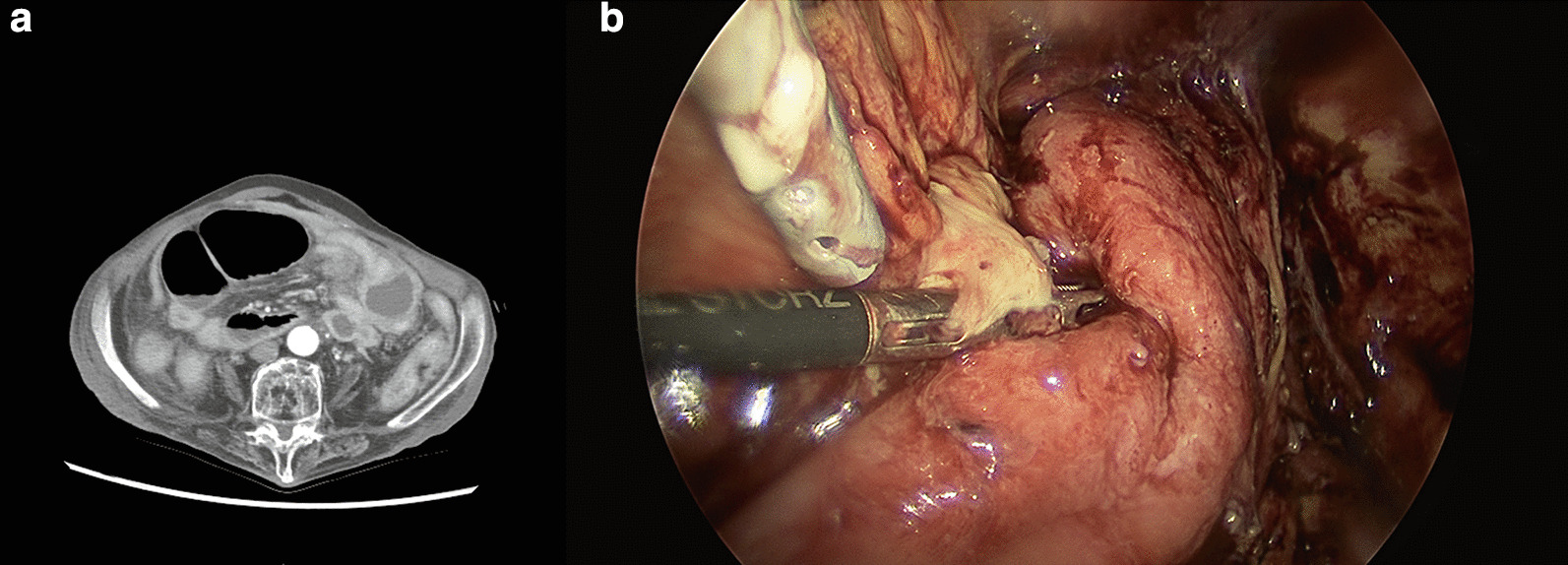
**a** CT scan of the patient. **b** A small intraabdominal abscess was found during surgery

The patient showed uneventful fever (38 ℃) and vomiting at postoperative day 18. A 3-cm-sized intraabdominal fluid collection was found at the left abdomen on CT scan (Fig. 4a), and she underwent emergency laparoscopic surgery. A small abscess was found and removed, and irrigation with saline was performed (Fig. 4b).

Small bowel obstruction occurred 4 days after the second surgery, and a Levin tube was inserted. She recovered without additional surgeries or adverse events and was discharged at 20 days after reoperation (38 days in total). Positron emission tomography, gastroscopy, and chest CT were performed to find the causes of elevated CEA before discharge, but no evidence of malignancy was found. She visited the outpatient clinic within one week and decided to use colostomy as a long-term approach.

## Discussion and conclusion

SP is very rare and occurs in about 0.9–1.1% of all colorectal surgeries reported at large institutions [2, 3]. The most common site of SP is the sigmoid colon, followed by the rectosigmoid colon, as it was first presented in 1894 [5–8]. Typical perforations have been reported due to the circular shape, are about 1 cm in diameter, and are located on the antimesenteric border of the colon [9]. SP mainly occurs in elderly patients with chronic constipation, but a few cases have been reported in younger patients [10–12]. Our patient was elderly and did not have a good performance status.

SP is an emergent condition that requires intraabdominal exploration, although the types of surgery vary and depend on several factors. If patient’s vital signs are stable and intraoperative findings are not severe, and anastomosis following resection of the perforated colon might be an option [12]. This approach can reduce discomfort and potential complications associated with colostomy, but anastomotic leakage can occur. However, most reports on SP have suggested that colostomy formation or exteriorization is needed to allow decompression of the proximal colon [1, 2, 5]. For the first admission, our patient underwent laparoscopic loop colostomy formation. The perforated site was exteriorized after mobilization because the proximal colon was dilated and filled with hard fecal material. Therefore, we focused on the possibility of salvage due to the unstable vital signs rather than primary anastomosis of the colon.

Definite predisposing factors for SP have not been established because it is a rare disease entity, and well-designed trials have not been published, with the exception of several case reports or series. However, in their systematic review, Chakravartty et al. suggested that risk factors for SP include chronic constipation, older age, nursing home residential status, fecal impaction, and increasing abdominal pain that is not explained by constipation alone [5]. Therefore, modifications of these factors should be attempted to try and avoid additional cases or causes for SP, even though our patient had a good status on discharge and acceptable outcomes. Colostomy takedown is a technically easy procedure that requires a short operation time. However, we recommend that physicians consider modification of the postoperative lifestyle before performing this procedure. There are currently no reports that have shown stercoral re-perforation after colostomy takedown, followed by colostomy formation for SP. Therefore, prognostic factors of immobility, a sequential organ failure assessment score, and lactate level suggested by Lee et al. might be helpful for assessing such patients [13].

Our patient was older (81 years old) and showed poorer activity and performance at the time of the second SP. Moreover, the frequency of defecation decreased compared with that at the time of first surgery. Severe peritonitis in the abdomen and postoperative adhesion were found during surgery and prolonged operation time during the second surgery compared with the first surgery. In addition, postoperative clinical course was poorer, including intraabdominal abscess and small bowel obstruction. Based on these findings, the authors and patient agreed against another colostomy takedown.

This report describes a case of stercoral re-perforation after colostomy takedown followed by colostomy formation for SP, which has important clinical considerations and serious implications. When the first colostomy formation was performed for SP, a decision about colostomy takedown should be carefully made, based on several factors. The patient’s quality of life is important but should be considered in the context of increasing patient risk for a life-threatening condition.

## Data Availability

All data generated or analyzed during this study are included in this published article.

## Abbreviations

CT: Computed tomography
SP: Stercoral perforation
